# Comparative genome analysis of VSP-II and SNPs reveals heterogenic variation in contemporary strains of *Vibrio cholerae* O1 isolated from cholera patients in Kolkata, India

**DOI:** 10.1371/journal.pntd.0005386

**Published:** 2017-02-13

**Authors:** Daisuke Imamura, Masatomo Morita, Tsuyoshi Sekizuka, Tamaki Mizuno, Taichiro Takemura, Tetsu Yamashiro, Goutam Chowdhury, Gururaja P. Pazhani, Asish K. Mukhopadhyay, Thandavarayan Ramamurthy, Shin-ichi Miyoshi, Makoto Kuroda, Sumio Shinoda, Makoto Ohnishi

**Affiliations:** 1 Collaborative Research Center of Okayama University for Infectious Diseases in India, Okayama University, Kolkata, India; 2 Department of Bacteriology I, National Institute of Infectious Diseases, Tokyo, Japan; 3 Pathogen Genomics Center, National Institute of Infectious Diseases, Tokyo, Japan; 4 Vietnam Research Station, Institute of Tropical Medicine, Nagasaki University, Nagasaki, Japan; 5 Division of Bacteriology, National Institute of Cholera and Enteric Diseases, Kolkata, India; 6 Translational Health Science and Technology Institute, Faridabad, India; 7 Graduate School of Medicine, Dentistry and Pharmaceutical Sciences, Okayama University, Okayama, Japan; Beijing Institute of Microbiology and Epidemiology, CHINA

## Abstract

Cholera is an acute diarrheal disease and a major public health problem in many developing countries in Asia, Africa, and Latin America. Since the Bay of Bengal is considered the epicenter for the seventh cholera pandemic, it is important to understand the genetic dynamism of *Vibrio cholerae* from Kolkata, as a representative of the Bengal region. We analyzed whole genome sequence data of *V*. *cholerae* O1 isolated from cholera patients in Kolkata, India, from 2007 to 2014 and identified the heterogeneous genomic region in these strains. In addition, we carried out a phylogenetic analysis based on the whole genome single nucleotide polymorphisms to determine the genetic lineage of strains in Kolkata. This analysis revealed the heterogeneity of the *Vibrio* seventh pandemic island (VSP)-II in Kolkata strains. The *ctxB* genotype was also heterogeneous and was highly related to VSP-II types. In addition, phylogenetic analysis revealed the shifts in predominant strains in Kolkata. Two distinct lineages, 1 and 2, were found between 2007 and 2010. However, the proportion changed markedly in 2010 and lineage 2 strains were predominant thereafter. Lineage 2 can be divided into four sublineages, I, II, III and IV. The results of this study indicate that lineages 1 and 2-I were concurrently prevalent between 2007 and 2009, and lineage 2-III observed in 2010, followed by the predominance of lineage 2-IV in 2011 and continued until 2014. Our findings demonstrate that the epidemic of cholera in Kolkata was caused by several distinct strains that have been constantly changing within the genetic lineages of *V*. *cholerae* O1 in recent years.

## Author summary

Seven cholera pandemics have been recorded throughout history, and the sixth, and presumably earlier pandemics, emerged from the Bay of Bengal. The seventh pandemic strain also appeared and spread from this area to different area of the world. Thus, the Bay of Bengal has always been considered the epicenter of cholera pandemics. In this report, we characterized the *V*. *cholerae* strains isolated from patients with cholera in Kolkata as a representative area of the Bay of Bengal between 2007 and 2014. The analysis revealed that the cholera epidemics were caused by several distinct *V*. *cholerae* O1 strains and that the predominant strains have genetically changed several times in recent years.

## Introduction

Cholera is an acute life-threatening diarrheal disease and remains a major health threat, particularly in developing countries in Asia, Africa, and Latin America [1,2]. It is estimated that 1.4 to 4.3 million cases of cholera and 28,000 to 142,000 deaths due to cholera, occur every year worldwide [3]. The Gram-negative bacterium *Vibrio cholerae* has more than 200 serogroups, but only O1 and O139 serogroups are responsible for epidemic and pandemic cholera [4,5]. Serogroup O1 has been further classified into two biotypes, classical and El Tor, based on several phenotypic traits. Each biotype has a unique nucleotide sequence within the genes encoding the cholera toxin responsible for severe diarrhea [4]. The cholera toxin is encoded on the lysogenic bacteriophage CTXΦ and consists of two subunits, A and B [6]. Corresponding amino acids at 20/39/68 of the cholera toxin B subunit of the classical biotype are H/H/T (*ctxB1* genotype), and of the El Tor biotype are H/Y/I (*ctxB3* genotype) [7]. Historically, seven cholera pandemics have been recorded since 1817. The sixth, and presumably earlier pandemics, emerged from the Bay of Bengal and were caused by the *V*. *cholerae* O1 classical biotype. However, the current seventh pandemic is caused by the El Tor biotype [5]. Recently, Hu et al. used comparative genomic analysis to demonstrate that the seventh pandemic strains originated from a nonpathogenic strain first observed in 1897 and slowly acquired virulence-associated elements by 1954 before becoming pandemic in 1961 [8]. Over the years, the *V*. *cholerae* O1 El Tor biotype has shown remarkable change and developed novel pathogenic variants that have the classical type *ctxB* gene (*ctxB1*) with an El Tor type genomic backbone [9–15]. Recently, the new *ctxB* variant (*ctxB7*) was found in Haiti, amongst other countries, which has N/H/T at the amino acid position 20/39/68 of the cholera toxin B subunit [16]. This newly appeared variant of *V*. *cholerae* has totally replaced the old strains indicating that the predominant strain has shifted during the current pandemic [16].

It has also been reported that the Haitian variant strain has evolved due to sequential events in the Indian subcontinent with some cryptic modification in the genome [17]. Mutreja et al. reported that the seventh pandemic strain first appeared in the Bay of Bengal and recurrently spread from this area to different parts of the world in at least three waves [18]. The Bay of Bengal is therefore considered the epicenter for the seventh cholera pandemic. It is important to monitor the appearance of new variants of *V*. *cholerae* in the Bay of Bengal, as it is possible that they will spread around the world in the future.

From the active diarrheal disease surveillance in the Infectious Diseases and Beliaghata General Hospital in Kolkata, it was established that *V*. *cholerae* O1 is one of the most common bacterial pathogens associated with diarrhea, with an estimated 11,000 cases every year [19]. Thus, cholera continues to be an important public health problem; hence, it is important to understand the genetic dynamism of *V*. *cholerae* from Kolkata, as a representative area of the Bengal region.

*Vibrio* Seventh Pandemic Island (VSP) was first detected by comparative genomic analysis of the classical and El Tor biotype strains of *V*. *cholerae* O1 [20]. Although the two genomic regions, VSP-I and VSP-II, were identified to be unique in the seventh pandemic El Tor strains, the role of these genomic islands in the pathogenicity of the organism is yet to be established. VSP-II is a 26.9-kbp genomic region composed of 24 genes between VC0490 and VC0516 according to the annotation of whole genome sequence (WGS) of *V*. *cholerae* N16961. These include genes encoding RNase, type IV pilin, chemotaxis, DNA repair, and transcriptional regulator [21]. Several variants of VSP-II have been reported in El Tor strains isolated from different continents, including Asia, Africa, and Latin America [22–26]. Therefore, characterization of VSP-II types is helpful in understanding the genetic lineages involved in the global transmission of cholera. In addition, WGS analysis is currently used as a powerful tool for understanding the various functional and evolutionary aspects of the organisms [27–32]. In this study, we carried out WGS analysis of *V*. *cholerae* O1 strains isolated between 2007 and 2014 from cholera patients in Kolkata in order to determine their genetic lineages. This analysis revealed the heterogeneity of VSP-II in Kolkata. In addition to the VSP-II genotype, phylogenetic analysis based on the whole genome single nucleotide polymorphisms (SNPs) revealed that shifts of predominant strains have occurred several times in recent years in Kolkata.

## Results

### Heterogeneous VSP-II

**Fig 1:**
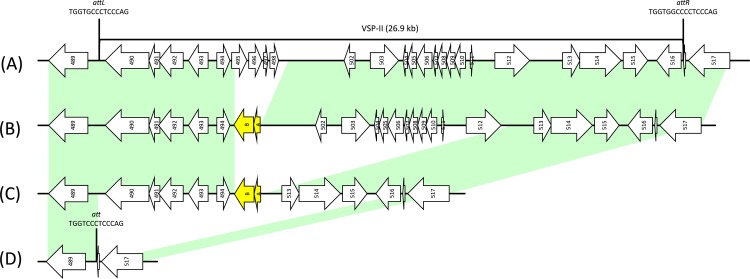
Genetic organization of VSP-II in *V*. *cholerae* O1 isolated from Kolkata, India. (A) Intact VSP-II of reference strain N16961, (B) VSP-IIB, (C) VSP-IIC, (D) VSP-IID of VSP-II negative strain. Arrows represent ORFs according to the annotation of *V*. *cholerae* N16961. Deduced sequence and location of *att* site are also shown.

To understand the genomic diversity of *V*. *cholerae* O1, we randomly selected 10 strains isolated from hospitalized cholera patients in each year between 2007 and 2014. Draft genome sequences of 80 strains were obtained using a next generation sequencer. Sufficient DNA sequencing reads were generated to cover the genome at least 60 folds in 79 strains. One strain was excluded from further analysis due to poor quality of the sequence data. The short sequence reads were mapped onto the genomic sequence of *V*. *cholerae* N16961 as an El Tor reference strain. We found that all strains lacked part of VSP-II, which is a 26.9-kbp genomic island consisting of 24 open reading frames (ORFs), VC0490 to VC0516 (Fig 1A). We determined the genetic organizations of VSP-II in these strains.

The region from 119th nucleotide of VC0495 to 1320 bp downstream of the VC0498 stop codon was replaced by a 1257 bp DNA fragment consisting of genes for transposase A and B subunits in 19 isolates (VSP-IIB, Fig 1B). Furthermore, 2 out of these 19 strains had an insertion of a 1767-bp DNA fragment of SXT IS4 family transposase gene at the 510th nucleotide of the VC0516 gene (VSP-IIBv1, S1 Fig) or at the 780th nucleotide of the VC0515 gene (VSP-IIBv2, S1 Fig).

Fifty-eight strains had a larger deletion than VSP-IIB. The 119th nucleotide of VC0495 to 596 bp downstream of the VC0512 stop codon (14,376 bp length) was replaced by genes for transposase A and B subunits (VSP-IIC, Fig 1C). Moreover, 9 of these strains had an insertion of the additional transposase gene fragment at the 560th nucleotide of VC0492 (VSP-IICv1, S1 Fig). The deleted regions are distinct in VSP-IIB and VSP-IIC; however, the upstream terminals of the replaced area (119th nucleotide of VC0495) were identical. These observations may suggest the existence of a hot spot for transposase.

Two strains were negative for VSP-II (VSP-IID, Fig 1D). The VSP-II was integrated between two attachment sites, *attL* (14 bp) and *attR* (16 bp), in all the VSP-II positive strains (Fig 1A). Murphy et al. demonstrated that VSP-II could be excised from the chromosome due to VC0516, which encodes an integrase, and the post-excision *att* site was of a shorter type (14 bp), identical to *attL* [32]. Classical strains O395 and V51, which are VSP-II-negative isolates, have a 16-bp *att* site identical to *attR*. However, VSP-II-negative strains in this study had an *att* site of 13 bp (Fig 1D). A shortened *att* site might be a consequence of excision of VSP-II from a VSP-II-positive El Tor strain.

Taken together, we identified three types of VSP-II in clinical *V*. *cholerae* O1 isolates in Kolkata (Fig 1). In addition, variants of VSP-IIC that have insertions of an additional transposase gene fragment in VC0492, and of VSP-IIB, that also has an insertion of SXT IS4 family transposase gene in VC0516 or VC0515, were identified (S1 Fig).

Frequencies of three VSP-II types in each year are shown in S2 Fig. Interestingly, VSP-IIC strains numbered less than half until 2009. This genotype rapidly spread in 2010 and eventually replaced the other types during subsequent years (S2 Fig). These results indicate that the genomic islands of *V*. *cholerae* O1 strains are frequently rearranged in Kolkata. VSP-IICv1 strains have an additional transposase gene fragment inserted into VC0492 (S1 Fig). Nine strains were identified as this variant, all of which were isolated in 2011, indicating that VSP-IICv1 strains appeared in 2011 and suddenly became predominant, but then quickly disappeared in 2012. The results also indicate that the predominant strain of *V*. *cholerae* O1 can rapidly shift in a particular area during an endemic, or that diverged strains were present in the environment of a particular area, which might have caused the seventh pandemic.

### *ctxB* variants

*V*. *cholerae* O1 El Tor strains were characterized by several *ctxB* variants including *ctxB3*, *ctxB1*, and *ctxB7* in the typical El Tor type, El Tor variant, and Haitian variant, respectively. Genotypes of *ctxB* and VSP-II of each strain used in this study are shown in S1 Table. Although strains were isolated from hospitalized patients with typical cholera symptoms, two strains were negative for *ctxB* (strain IDH-00115 in 2007 and IDH-02185 in 2009). Our hospital-based surveillance screened for 25 enteric pathogens including 15 bacteria, 6 viruses and 4 parasites in each fecal sample [19]. However, the *ctxB* negative *V*. *cholerae* O1 was the sole pathogen detected. Although epidemic cholera is caused by cholera toxin-positive *V*. *cholerae*, strains without cholera toxin can cause a diarrheal disease through other possible virulence factors, including the heat-stable toxin (NAG-ST) [33], hemolysin (Hly), type III secretion system (T3SS) [34, 35], cholix toxin (Chx) [36, 37], mannose sensitive hemagglutination (MshA) and repeat in toxin (RtxA). Two *ctxB*-negative strains, as well as the *ctxB*-positive strain in current study, harbored genes encoding for Hly, MshA and RxtA, but not NAG-ST, structural proteins of T3SS and Chx. The other 77 strains harbored the *ctxB* gene, either *ctxB1* (n = 20) or *ctxB7* (n = 57) genotypes. As shown in S1 Table, 18 strains with *ctxB1* had VSP-IIB and the other 2 strains had VSP-IID. All 57 strains with *ctxB7* had VSP-IIC. Both VSP-II and CTXΦ prophage are mobile elements and the distance between these elements is more than 1 Mbp on the 2.96 Mbp on chromosome 1 in the reference genome. If the mutations in *ctxB* and VSP-II were independent events, either element may have affected the acquisition or selectivity of another element.

### Phylogenetic analysis

**Fig 2:**
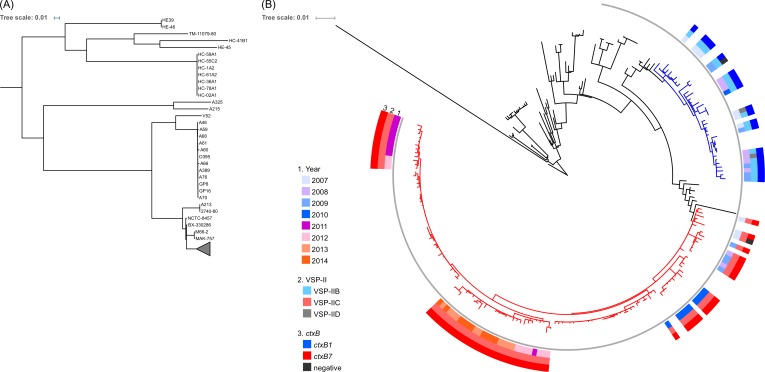
Maximum likelihood tree generated from SNPs in the core genome. (A) Phylogenetic tree of the 7th pandemic El Tor strains with the non-O1, non-O139 and pre-seventh pandemic strains. 100,600 SNPs were identified in 276 strains. Gray triangle show collapsed clade containing the 7th pandemic El Tor strains. (B) Phylogenetic tree of the 7th pandemic clade containing all Kolkata isolates. 1,772 SNPs were identified in 243 strains. Branches colored blue and red indicate lineage 1 and 2, respectively. Gray thin circle show wave 3 lineage. Positions of the Kolkata isolates are indicated with color rings representing their characteristics on the outside tree.

**Fig 3:**
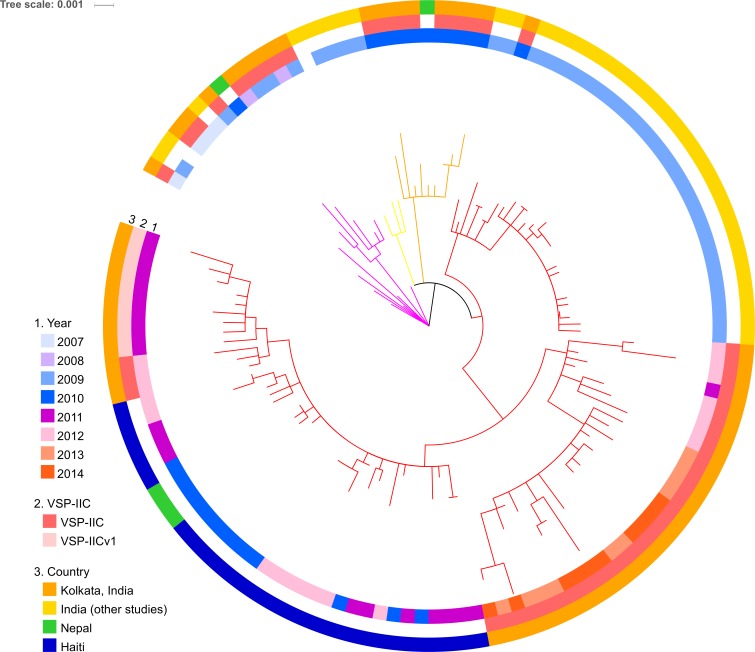
Phylogenetic sub-tree of strains in lineage 2. Magenta, yellow, orange, and red branches represent sublineage 2-I, 2-II and 2-III, respectively. Other characteristics of strains are indicated by the three colored rings surrounding the tree. Information of VSP-II type was obtained only from Kolkata isolates.

To assess the genetic lineage of *V*. *cholerae* O1 clinical isolates in Kolkata, we performed phylogenetic analysis based on the genome-wide SNPs. As shown in Fig 2A, the seventh pandemic El Tor strains clade differed from the pre-seventh pandemic strains. The 79 *V*. *cholerae* strains isolated in Kolkata between 2007 and 2014 belonged to wave 3 of seventh pandemic and were classified into two lineages (Fig 2B, blue and red branches). Lineage 1 contained 20 Kolkata strains with the *ctxB1* allele, one Kolkata strain negative for *ctxB*, and other strains isolated in India and Nepal. In addition to 19 VSP-IIB strains, the two VSP-IID strains were also found in this lineage. This result is consistent with the notion that these VSP-IID strains are not of the classical biotype but instead seventh pandemic strains after the excision of VSP-II, as suggested by the short *att* site. Each VSP-IID strain is closely related to each other and with the VSP-IIB strains, suggesting that VSP-IID is a derivative of the VSP-IIB strain as a consequence of the excision of VSP-II. Lineage 2 contains 58 strains, of which 57 strains possesses *ctxB7* and one negative for *ctxB* gene. Although all 58 lineage 2 strains harbored VSP-IIC, lineage 2 was divided into four sublineages, I, II, III, and IV (Fig 3). Lineages 2-I, 2-II, and 2-III comprised South Asian isolates; however, 2-IV also contained Haitian isolates. In this lineage, Kolkata strains isolated between 2010 and 2014 were more clustered among themselves owing to the relatedness between Nepalese and Haitian isolates (Fig 3). Strains with VSP-IICv1, which is a transposon-inserted variant of VSP-IIC, formed a cluster (Fig 3), suggesting clonal expansion of the lineage 2-IV with VSP-IICv1. Each of the two *ctxB*-negative strains was phylogenetically within lineages 1 and 2, therefore seeming to emerge independently.

### Temporal analysis

**Fig 4:**
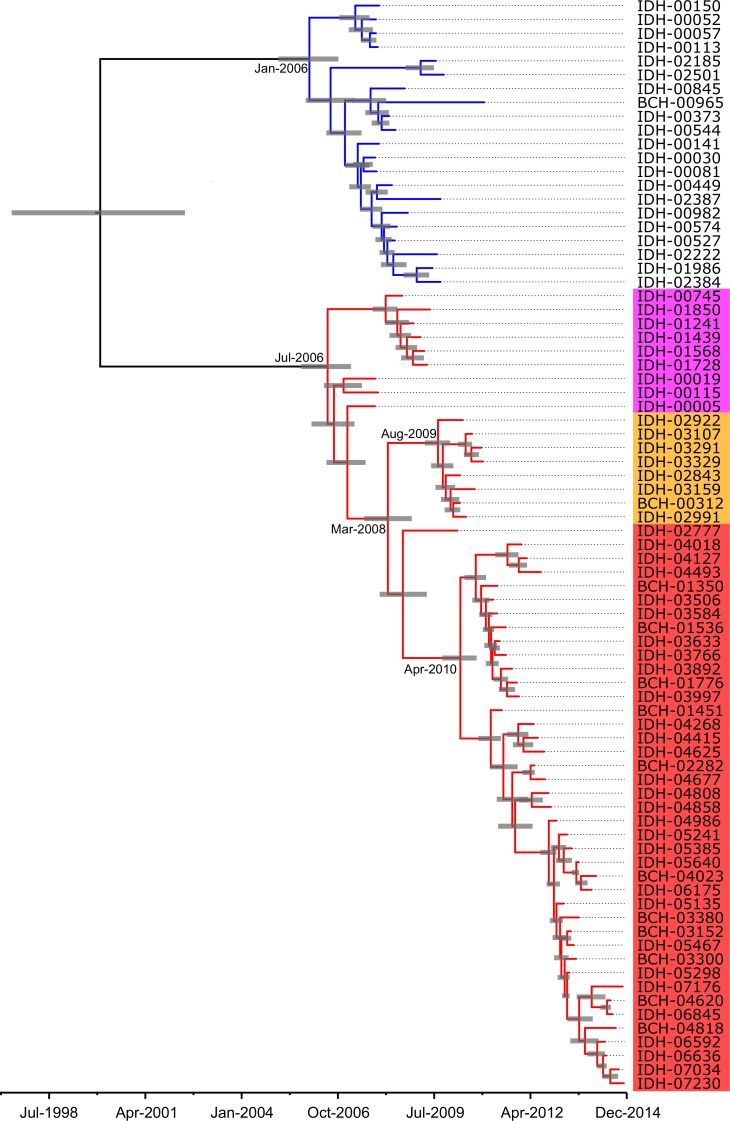
Maximum clade credibility tree of *V*. *cholerae* O1 isolates in Kolkata. Branches colored blue and red indicate lineage 1 and 2, respectively. Node bars represent the 95% highest posterior density (HPD) range for the estimated divergence time at each node. Background of strain is colored according to the respective sublineage in Fig 3.

Phylodynamic analysis of the 79 Kolkata strains was investigated via Bayesian analysis (Fig 4). Kolkata strains between 2007 and 2014 were divided into lineage 1 (n = 21), which possesses VSP-IIB and *ctxB1*, and lineage 2 (n = 58) that largely possesses VSP-IIC and *ctxB7*. It was estimated from the maximum clade credibility tree that the most recent common ancestor (MRCA) of lineage 2 emerged in July 2006 (95% HPD: September 2005 to February 2007). Lineage 1 emerged in January 2006 (95% HPD: January 2005 to October 2006) and the isolates in lineage 1 lasted until December 2010. Consequently, two distinct *V*. *cholerae* O1 lineages were concurrently distributed in Kolkata between July 2006 and December 2010. As for the 58 strains in lineage 2, Kolkata strains belonged to lineage 2-I (n = 9), 2-III (n = 8), or 2-IV (n = 41). The isolates of lineage 2-I that circulated between 2007 and 2009 were replaced with the isolates of lineage 2-III. The analysis predicted that the MRCA of lineage 2-III existed in August 2009 (95% HPD: April 2009 to December 2009); however, lineage 2-III was transient, at least in Kolkata, because the isolates were only observed in 2010. Lineage 2-IV strains followed the transient lineage 2-III spike in Kolkata, for which the divergence time of the two lineages was estimated to be in March 2008 (95% HPD: July 2007 to November 2008). All 40 strains between 2011 and 2014 belonged to the lineage 2-IV cluster with Nepalese and Haitian isolates, and the MRCA seemed to emerge in April 2010 (95% HPD: September 2009 to September 2010). The first lineage 2-IV strain isolated in March 2010 was different from the other lineage 2-IV Kolkata strains and assigned to a distinct cluster of Indian isolates, which were isolated in Northern India in 2009 [23], suggesting that a surge in lineage 2-IV including Haitian isolates in Kolkata started suddenly at April 2010 and continued until 2014.

## Discussion

WGS of clinical *V*. *cholerae* O1 isolates in Kolkata determined the sequence variation of VSP-II, which related to the *ctxB* allele. The phylogenetic analysis of strains found in Kolkata revealed two distinct lineages (lineages 1 and 2) and the coexistence of strains of both lineages between July 2006 and December 2010, thus indicating the concurrent prevalence of at least two genetically distinct *V*. *cholerae* strains. However, the ratio of the two lineages changed markedly from 2010 onward. Lineage 2 strains increased in 2010 and totally replaced lineage 1 strains in 2011, which continued to be predominant until 2014. Additionally, strains in lineage 2 were diverse and showed a temporal pattern. Lineage 2 strains isolated between 2007 and 2009 were categorized as lineage 2-I and those observed during 2010, lineage 2-III. Strains in lineage 2-IV were first found in 2010 and then became predominant. Around the same time elsewhere in India, *V*. *cholerae* strains showed variations in several genes and seemed to evolve sequentially with some cryptic modification in the genome [17, 38, 39]. Our results are in agreement with previous findings of genome-wide SNP analysis, suggesting that the genotypes of *V*. *cholerae* O1 in Kolkata had been replaced on several occasions in recent years.

Since the discovery of the prototypical VSP-II genomic island in 2004 [21], several variants have been identified from different continents. An environmental isolate in Brazil in 1982, TMA21, had deletions from downstream of VC0498 to VC0503 and from VC0511 to VC0515 [26]. Clinical isolates in Peru between 1991 and 2003 lacked genes VC0512 to VC0515 [25], and West African and South American isolates between 1981 and 1985 also lacked VC0512 to VC0515 [18]. In Africa, Zambian isolates from 2003 to 2004 had deletions from VC0493 to VC0498 [23]. However, such VSP-II variants were not found in Kolkata between 2007 and 2014. CIRS101 isolated in Bangladesh in 2002 had a substitution from VC0495 to VC0512 by transposase, which is identical to VSP-IIC in this study [26]. Moreover, both VSP-IIB isolates and VSP-IIC isolates were found in Chandigarh, a province of northern India, in 2009 [22]. These strains could emerge and spread widely throughout the Indian subcontinent and, further work with retrospective analysis would be required to elucidate the emergence mechanism of VSP-II variants. It has been reported that *V*. *cholerae* strains in all pandemics disseminated from the Bay of Bengal to the rest of the world [18] and considering this tendency, it is possible that the new VSP-II variants could spread beyond this region.

Taviani et al. reported that among 97 isolates in Bangladesh between 2004 and 2007, 96 strains harbored VSP-IIC [26]. This type was found to be predominant in Kolkata after 2010 (S2 Fig). Although both Kolkata and Bangladesh border the Bay of Bengal, transition patterns of predominant strains are temporally distinct. In addition, prevalence of the *ctxB* allele also differed between Dhaka, Bangladesh, and Kolkata. In Dhaka, isolates with *ctxB1* reemerged in 2012 and became dominant between 2013 and 2014 by outcompeting the former dominant *ctxB7* strains [40]. During the same period in Kolkata, all strains possessed the *ctxB7* allele [17] and belonged to lineage 2-IV in this study. From our SNP analysis and correlation with *ctxB* typing, we speculate that the current predominant strain in Dhaka belongs to lineage 1. Considering the prevalence trends of *V*. *cholerae* O1 in Kolkata, novel genetic variants may appear frequently and spread to other regions.

Our genome-wide SNP analysis demonstrates the phylogenetic relatedness between Kolkata strains and strains isolated from other areas, especially strains in lineage 1. However, the lineage 2 strains formed a spatiotemporal homogeneous cluster. The limited amount of available genome data might affect the apparent homogeneous cluster formation. Two exceptions are observed in clusters in lineage 2-III, formed by Kolkata and Nepalase strains, and in lineage 2-IV. The former, a cluster made by 8 Kolkata strains isolated in 2010 includes one Nepalese 2010 strain. The other cluster, consisting of Northern India strains isolated in 2009, includes one Kolkata strain isolated in 2010. More genomic data from next-generation sequencing would reveal more precise dissemination and evolutionary trends of *V*. *cholerae* O1. WGS-based analysis could help us to understand the temporal and geographical spread of *V*. *cholerae*; hence, continued monitoring of *V*. *cholerae* O1 is needed in all cholera endemic regions. In addition, WGS has been utilized in several studies to understand global transmission and phylogeny of pathogenic bacteria including *V*. *cholerae*, *Shigella dysenteriae* and *Salmonella* Enteritidis [18, 41, 42]. Our work characterized the transition of predominant strains during several continuous years at the epicenter of cholera. Combining these studies with computational modeling may enable us to predict strains that cause epidemics throughout the world.

## Methods

### Bacterial strains

*Vibrio cholerae* O1 strains used in this study are listed in S1 Table with the year and month of isolation. These strains were isolated from fecal samples of hospitalized patients with typical cholera symptoms in Kolkata, India between 2007 and 2014. *V*. *cholerae* strains were isolated by streaking the stool samples on thiosulphate citrate bile salts sucrose (TCBS) agar plates and typical sucrose fermenting yellow colonies were tested by serum agglutination using *V*. *cholerae* O1 polyvalent antiserum (Becton Dickinson, Sparks). Isolated strains were stored in -80°C as glycerol stock. Fecal samples were collected two days a week from every fifth diarrheal patient (approximately 5.7% of total diarrheal patients). Annually, 93 to 363 samples were positive for *V*. *cholerae* O1 between 2007 and 2014. Ten *V*. *cholerae* O1 strains were chosen each year at random to represent predominant months of each year and subjected to the analysis. The patients were aged between 1 month and 89 years at two hospitals in Kolkata, and all patients were discharged after treatment.

### Whole-genome sequencing

Genomic DNA was prepared using the DNeasy Blood & Tissue Kit (Qiagen) according to the manufacturer’s instructions. In total, 80 *V*. *cholerae* O1 strains were used for whole genome sequencing. We prepared Illumina libraries using Nextera XT DNA Library Preparation Kit (Illumina) and sequenced paired-end Illumina short reads for each library on HiSeq 2500 (Illumina) or MiSeq (Illumina) sequencers. The sequence reads were mapped with *V*. *cholerae* O1 El Tor reference strain N16961 by using CLC Genomics Workbench 8.5.1 (CLC bio) to obtain whole genome alignment. The reads were assembled using a de novo genome assembly program of CLC Genomics Workbench and a multicontig draft genome was generated for each sample. Nucleotide sequence data were submitted to the DDBJ Sequenced Read Archive and each accession number is listed in S1 Table.

### Identification of VSP-II

Comparison of short sequence reads with the genomic sequence of *V*. *cholerae* N16961, a prototype strain of seventh pandemic El Tor biotype, suggested the lack of a large area in the VSP-II genomic island; thus, genetic organizations of VSP-II in each strain used in this study were identified (Fig 1). Among 79 strains, 19, 58, and 2 were found to lack the ORFs from VC0495 to VC0498, VC0495 to VC0512, and the entire VSP-II sequence, respectively. Strains lacking the entire VSP-II sequence generated a contig cover from upstream to downstream of VSP-II region (Fig 1D).

Nineteen strains lacked the internal region of VSP-II from VC0495 to VC0498 as compared with the N16961 sequence and did not generate a contig cover from upstream to downstream of the missing area. This area of each strain was PCR amplified and the sequence of this amplicon was determined using the Sanger method (Fig 1B). Among these 19 strains, 2 strains had truncated contigs within VSP-II region in addition to the lacking area. These contigs were also amplified and the sequence was determined (S1 Fig).

The rest of the 58 strains were found to lack the ORFs from VC0495 to VC0512 as compared with the N16961 sequence. This area of each strain was PCR amplified and determined the sequence of a representative strain (Fig 1C). To confirm the identity of VSP-II, the entire VSP-II fragments of these strains were amplified and analyzed by Restriction Fragment Length Polymorphism (RFLP) using *Bgl*II. Nine strains showed altered band patterns compared with the others, and the difference of these VSP-II sequence were identified by PCR amplification and sequencing (S1 Fig).

### SNP detection and phylogenetic analysis

To remove adapter sequences and low quality bases with a Phred score of less than 15 from the short reads, read trimming was performed using fastq-mcf (https://expressionanalysis.github.io/ea-utils/) and sickle (https://github.com/najoshi/sickle) program. Simulated paired-end reads were constructed from the available genomic sequences of *V*. *cholerae* strains using SimSeq software [43] with the following parameter: number of pairs of reads, ‘‘read_number 2000000”; mean library insert size, ‘‘insert_size 150”; and paired-end reads length of 120 mer, ‘‘21 120 22 120”. These parameters indicated that 4 million hypothetical 120-mer reads were generated without mutations or indels from the genomic sequences used for SNP identification. The trimmed or simulated reads with at least 40 mer were mapped using the BWA-mem program [44] against the N16961 sequence (NC_002505.1 and NC_002506.1) [45], and the read mapping data were constructed by the samtools program [46]. All SNPs with at least a 5× coverage depth and a Phred score of at least 20 were extracted using VarScan v2.3.4 [47]. The SNPs on the repeat and prophage regions of the N16961 genome, which was identified by the NUCmer [48] and PHAST [49] program, was excluded for further core-genome phylogeny analysis. Additionally, to exclude for the recombination regions, RecHMM was used to identify the recombination region [50]. The remaining SNPs were concatenated to generate a pseudo sequence for phylogenetic analysis; maximum likelihood phylogenetic analysis was performed using RAxML v8.2.0 [51] with 1,000 bootstrap iterations. The trees were visualized using iTOL 3 (http://itol.embl.de/) [52].

### Bayesian phylogenetic inference

To estimate the divergence date of the *V*. *cholerae* O1 isolate in Kolkata, we performed a temporal analysis using the Bayesian Evolutionary Analysis Sampling Trees (BEAST) v.2.4.4 software package [53]. The isolation date of each strain was used as tip data. A random clock model was implemented using Markov Chain Monte Carlo (MCMC) chains run for 100 million generations with 10% burn-in and sampled every 1000 generations. A GTR nucleotide substitution model was used with a gamma distribution with four rate categories. The effective sample sizes were greater than 200 for all estimated parameters and tree data were summarized to generate the maximum clade credibility tree.

### Ethics statement

This study was approved by the duly constituted Institutional Ethics Committee (IEC) of National Institute of Cholera and Enteric Diseases. As per the recommendation of IEC, individual written informed consent was obtained from each adult patient and a parent or guardian of child patient enrolled in this study and confidentiality was maintained.

## Supporting information

S1 Table Characterization of *V*. *cholerae* O1 clinical isolates in Kolkata. (XLSX)

S2 Table List of genomes from public databases. (XLSX)

S1 Fig Genetic organization of VSP-IIB and VSP-IIC variants. Arrows represented ORF according to the annotation of *V*. *cholerae* N16961.
(TIF)

S2 Fig Temporal shift of VSP-II type distribution in Kolkata, India from 2007 to 2014. (TIF)
