# Supplementary figures and images for: Comparative genome analysis of VSP-II and SNPs reveals heterogenic variation in contemporary strains of Vibrio cholerae O1 isolated from cholera patients in Kolkata, India
Source: PLoS Negl Trop Dis. 2017 Feb 13;11(2):e0005386. doi: 10.1371/journal.pntd.0005386 (PMC5349696; doi:10.1371/journal.pntd.0005386)

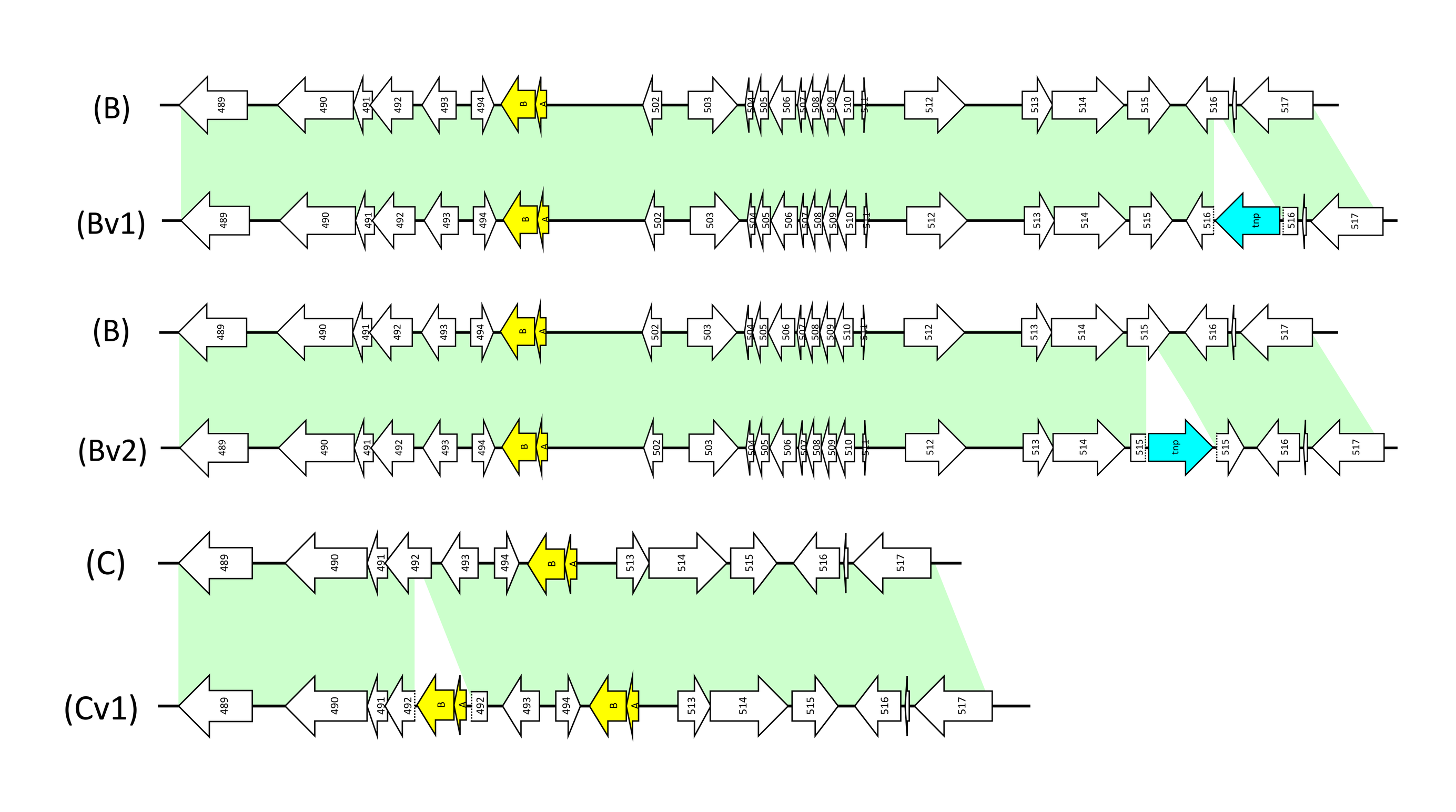

Supplement: S1 Fig — Arrows represented ORF according to the annotation of V. cholerae N16961. (TIF) [file pntd.0005386.s003.tif]

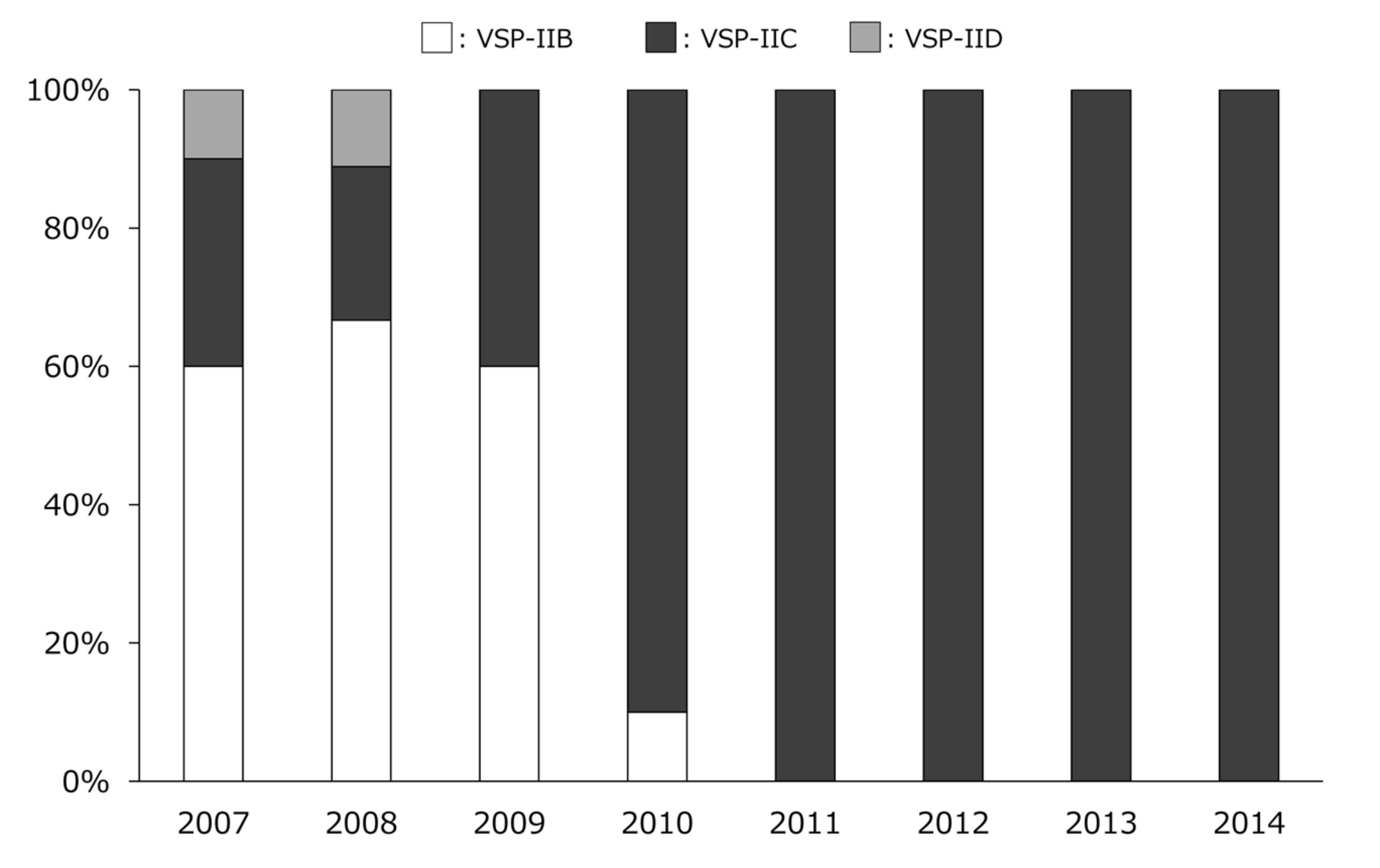

Supplement: S2 Fig — (TIF) [file pntd.0005386.s004.tif]
